# Long‐distance natal dispersal is relatively frequent and correlated with environmental factors in a widespread raptor

**DOI:** 10.1111/1365-2656.13272

**Published:** 2020-07-13

**Authors:** Hanna M. McCaslin, T. Trevor Caughlin, Julie A. Heath

**Affiliations:** ^1^ Department of Biological Sciences Boise State University Boise ID USA; ^2^ Raptor Research Center Boise State University Boise ID USA; ^3^ Department of Fish, Wildlife, and Conservation Biology Colorado State University Fort Collins CO USA

**Keywords:** agriculture, American kestrel, bird banding, *Falco sparverius*, migratory strategy, sex‐biased, weather

## Abstract

1. Dispersal is a critical process influencing population dynamics and responses to global change. Long‐distance dispersal (LDD) can be especially important for gene flow and adaptability, although little is known about the factors influencing LDD because studying large‐scale movements is challenging and LDD tends to be observed less frequently than shorter‐distance dispersal (SDD).
2. We sought to understand patterns of natal dispersal at a large scale, specifically aiming to understand the relative frequency of LDD compared to SDD and correlates of dispersal distances.
3. We used bird banding and encounter data for American kestrels (*Falco sparverius*) to investigate the effects of sex, migration strategy, population density, weather, year and agricultural land cover on LDD frequency, LDD distance and SDD distance in North America from 1961 to 2015.
4. Nearly half of all natal dispersal (48.9%) was LDD (classified as >30 km), and the likelihood of LDD was positively associated with the proportion of agricultural land cover around natal sites. Correlates of distance differed between LDD and SDD movements. LDD distance was positively correlated with latitude, a proxy for migration strategy, suggesting that migratory individuals disperse farther than residents. Distance of LDD in males was positively associated with maximum summer temperature. We did not find sex‐bias or an effect of population density in LDD distance or frequency. Within SDD, females tended to disperse farther than males, and distance was positively correlated with density. Sampling affected all responses, likely because local studies more frequently capture SDD within study areas.
5. Our findings that LDD occurs at a relatively high frequency and is related to different proximate factors from SDD, including a lack of sex‐bias in LDD, suggest that LDD may be more common than previously reported, and LDD and SDD may be distinct processes rather than two outcomes originating from a single dispersal distribution. To our knowledge, this is the first evidence that LDD and SDD may be separate processes in an avian species, and suggests that environmental change may have different outcomes on the two processes.

## Graphical abstract

The authors assess the relative frequency and correlates of shorter‐distance dispersal (SDD) and long‐distance dispersal (LDD) natal dispersal using banding data. LDD was more frequent than previously reported and correlates of LDD and SDD differed. LDD and SDD may be distinct processes, rather than a single right‐skewed dispersal kernel, and environmental change may influence the two movements differently.

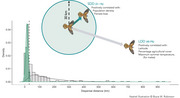

## Resumen

1. La dispersión es un proceso crítico que influye en la dinámica de poblaciones y cómo éstas responden al Cambio Global. La dispersión a larga distancia (LDD) puede ser especialmente importante para el flujo de genes y la adaptabilidad, sin embargo se conoce poco sobre los mecanismos que subyacen a la LDD, ya que su estudio a largo plazo es difícil, lo que resulta en una menor frecuencia de observación de LDD en comparación con la dispersión a corta distancia (SDD).
2. Buscamos comprender los patrones de dispersión natal a gran escala, en particular, comparar la frecuencia relativa de LDD versus SDD, así como la correlación entre las distancias de dispersión y diversos factores.
3. Utilizamos datos de anillamiento y avistamiento del Cernícalo Americano (*Falco sparverius*) para investigar los efectos del sexo, estrategia de migración, densidad poblacional, meteorología, año, y cobertura de tierra agrícola sobre la frecuencia de LDD, y la distancia de LDD y SDD en Norte América para el período 1961–2015.
4. Casi la mitad de la dispersión natal (48.9%) fue LDD (definida como distancia >30 km), y la probabilidad de LDD estuvo positivamente asociada con la proporción de tierra agrícola alrededor de los sitios de cría. Las correlaciones de las distancias fueron diferentes para los desplazamientos LDD y SDD. La distancia LDD estuvo positivamente correlacionada con la latitud, un indicador de la estrategia de migración, lo que sugiere que los individuos migratorios se dispersan más que los residentes. La distancia LDD en machos estuvo positivamente asociada con la temperatura máxima de verano. No encontramos sesgo en el sexo ni efecto de la densidad poblacional sobre la distancia y frecuencia de LDD. Respecto a SDD, las hembras tendieron a dispersarse más que los machos, y la distancia estuvo positivamente correlacionada con la densidad. El muestreo afectó a todas las variables de respuesta analizadas, probablemente debido al hecho de que los estudios a escala local detectan con mayor frecuencia SDD dentro de las áreas de estudio.
5. Nuestro hallazgo referente al hecho de que LDD sucede con relativa alta frecuencia y está controlada por factores diferentes a los de SDD, como por ejemplo, la ausencia de sesgo debido al sexo en LDD, sugiere que LDD puede ser más común de lo propuesto hasta ahora, y que LDD y SDD pueden ser considerados como dos procesos distintos y no como resultados procedentes de una única distribución de dispersión. Hasta donde sabemos, ésta es la primera evidencia de que LDD y SDD pueden ser dos procesos separados en aves, sugiriendo que los cambios ambientales pueden afectarles de manera diferente.

## 1 INTRODUCTION

Dispersal occurs in nearly all organisms and is the primary mechanism of gene migration between populations (Clobert, Danchin, Dhondt, & Nichols, 2001). Dispersal influences individual fitness, population genetic structuring and diversity, and is a key factor in species' abilities to adapt to global change (Hanski & Gilpin, 1997; Kokko & López‐Sepulchre, 2006). Thus, understanding how individuals integrate and respond to the intrinsic and environmental factors underlying dispersal is important for understanding population dynamics and potential species' responses to global change.

Natal dispersal, defined as the movement between natal area and the area where first breeding takes place (Clobert et al., 2001), is common and tends to occur over greater distances than dispersal between breeding locations by adults (Greenwood & Harvey, 1982). The majority of natal dispersal movements occur at relatively short distances with some movements reaching longer distances so that the distributions of natal dispersal tend to be right‐skewed and heavier‐tailed than normal distributions (Nathan, 2006). Long‐distance dispersal (LDD) movements are often viewed as stochastic outliers (Nathan, 2006) and this, coupled with the logistical challenges of studying long‐distance animal movements (Koenig, Van Vuren, & Hooge, 1996), has led most animal dispersal studies to occur at scales smaller than the full dispersal distribution. However, small‐scale studies can result in biases towards short‐distance dispersal (SDD) movements and often underestimate or fail to detect LDD (Morton et al., 2018). Bias towards SDD events may lead to an incomplete understanding of the causes and consequences of natal dispersal. For example, compared to SDD, LDD can have disproportionate effects on gene flow, connectivity and species persistence (Goldwasser, Cook, & Silverman, 1994; Tittler, Fahrig, & Villard, 2006). It is also unclear whether the ultimate causes of dispersal, inbreeding avoidance, competition for food or breeding sites and matching habitat to phenotype (Bowler & Benton, 2005; Clobert et al., 2001; Edelaar, Siepielski, & Clobert, 2008) affect SDD and LDD equally. Therefore, a better understanding of the frequency of LDD and the proximate correlates of SDD and LDD distance is needed.

Intrinsic and extrinsic factors influence animal dispersal patterns within and among taxa, affecting both tendency and magnitude of dispersal movements (Clobert, Baguette, Benton, & Bullock, 2012). Body size and diet correlate with natal dispersal distance in birds and mammals (Sutherland, Harestad, Price, & Lertzman, 2000) and dispersal is often sex‐biased, with males dispersing farther than females in many mammal species and females dispersing farther than males in most bird species (Greenwood, 1980). In birds, dispersal distance is positively correlated with migration distance within a single species (Kelly et al., 2016), and migratory songbird species tend to disperse farther than non‐migratory species (Paradis, Baillie, Sutherland, & Gregory, 1998). Environmental factors including habitat type influence dispersal rate (Berry, Tocher, Gleeson, & Sarre, 2005), and this effect may be intensified by intrinsic factors, if it is more difficult for individuals with poorer physical condition to move through certain habitats (del Mar Delgado, Penteriani, Revilla, & Nams, 2010). Additionally, natal dispersal distance is positively correlated with temperature during the post‐fledging period in late summer and early fall in common buzzards (*Buteo buteo*), suggesting that warm temperatures create favourable conditions for flight dynamics and movement (Walls, Kenward, & Holloway, 2005). Similarly, natal dispersal distance in Arctic terns (*Sterna paradisaea*) is positively correlated with temperature during dispersal and in the previous breeding season, suggesting temperature directly affects dispersal movement and indirectly influences dispersal through maternal care and provisioning (Møller, Flensted‐Jensen, & Mardal, 2006). Finally, environmental conditions that influence resource availability or nesting success can affect dispersal rates by altering population density, which is another important determinant of dispersal propensity (Matthysen, 2005). For example, in white‐throated dippers (*Cinclus cinclus*), warm winters increase overwintering survival and conspecific densities leading to a higher rate of LDD (Sæther et al., 2000).

The American kestrel (*Falco sparverius*) is a widespread species that breeds throughout North America, and populations display continuous variation in migratory strategies along a latitudinal cline, from fully resident southern populations to fully migratory northern populations (Smallwood & Bird, 2002; Smallwood, Causey, et al., 2009). Recent genetic work on kestrels shows that migratory populations have low genetic structure compared to resident populations (Miller, Mullins, Parrish, Walters, & Haig, 2012). This pattern suggests that migration and dispersal distances may be positively correlated. Kestrels frequently nest near agricultural areas that are open landscapes suitable for hover hunting and have high abundances of prey species like small mammals and insects (Shave & Lindell, 2017; Smallwood, 1987; Smallwood, Winkler, Fowles, & Craddock, 2009; Touihri, Séguy, Imbeau, Mazerolle, & Bird, 2019). For several decades, American kestrels have been captured and marked via nest box projects and numerous studies have addressed short‐distance kestrel dispersal within project areas (Table 1). These studies show kestrels display female‐biased dispersal, in which females may disperse nearly twice as far as males, and median dispersal distances are approximately 7 km (Smallwood & Bird, 2002; Steenhof & Heath, 2013). However, studies of kestrel recruitment (Steenhof & Heath, 2013) and demography (Brown & Collopy, 2013, C.J.W. McClure, unpubl. data) suggest extensive external recruitment and indicate that long‐distance dispersal contributes to stable populations. This suggests that local nest box studies of dispersal may not represent the frequency and magnitude of LDD in kestrels.

**Table 1:** Summary of American kestrel natal dispersal studies conducted within study areas with nest boxes and this study based on banding and encounter data. In previous studies, the majority of kestrel individuals dispersed short distances, but these studies have limited potential to detect long‐distance movements resulting in settlement outside of the study area

	Median dispersal distance (km)		Maximum dispersal distance (km)		Sample size	Study area size (km^2^)
	Male	Female	Male	Female		
Jacobs (1995)	16.0	30.0	—	—	10	75
Miller and Smallwood (1997)^a^	4.4	5.1	32.4	38.8	34	1,200
Steenhof and Heath (2013)	3.5	8.1	24.1	42.9	81	1,000
This study	23.5	33.7	938.3	772.6	311	Continental

^a^ Subspecies *Falco sparverius paulus*

Our objective was to examine natal dispersal distance and direction using data collected at a larger scale than previous studies. Specifically, we were interested in the relative frequency of dispersal distances longer than the maximum distances recorded in nest box studies, and the correlates of LDD and SDD movements recorded in banding records. We hypothesized that long‐distance natal dispersal frequency and distance in kestrels could be explained by a combination of intrinsic and environmental factors, but that these factors may differ from the correlates of SDD movements. We predicted that frequency and distance of long‐distance dispersal would be female‐biased, migratory individuals would disperse farther than non‐migratory individuals, and individuals from natal areas with high population density would be more likely to exhibit LDD. Also, we predicted that temperatures during key phases of the annual cycle would correlate with dispersal distance, specifically that maximum temperatures during hatching and post‐fledging exploration would be positively correlated with distance if nestling physical condition affects dispersal distance, and if dispersal occurs during the exploratory post‐fledging phase, respectively, and that minimum temperatures during either winter or nest establishment would be negatively correlated with dispersal distance if migration and dispersal distance phenotypes are correlated or if natal dispersal occurs in the spring following birds' first winter. We expected that percentage of agriculture would be negatively correlated with long‐distance dispersal frequency and distance because agriculture may be high‐quality habitat for kestrels, and that temperature and agricultural changes over time would lead to temporal trends in dispersal distance. Males and females may respond differently to environmental conditions because of the drivers of sex‐biased dispersal, so we predicted that sex may interact with environmental factors including temperature and percentage of agriculture. Additionally, we predicted that migratory strategy and temperature would interact to cause individuals from higher latitudes to increase dispersal distance more over time than those at lower latitudes. Finally, we expected that correlates of SDD would be similar to those found in local studies, and females would disperse farther than males, individuals from higher latitudes would disperse farther than those from lower latitudes, and individuals from areas with relatively higher percentage of agriculture would disperse relatively shorter distances.

## 2 MATERIALS AND METHODS

We obtained banding and encounter data from the US Geological Survey's Bird Banding Laboratory for all kestrels banded in the United States or Canada from 1961 to 2015. As of May 2017, 329,987 kestrels were reported banded during this timeframe, and 5,329 (1.6%) of those birds were subsequently encountered (alive or dead) and reported by scientists or the public. We defined natal dispersers as ‘local’ (nestling) or ‘hatch year’ birds banded during the breeding season (1 April–15 August) and encountered during the breeding season 1 year later. We assumed that birds encountered during this period were within their breeding territory because approximately 85% of kestrels breed in their second year (Steenhof & Heath, 2009). We removed all birds with any of the following in either the banding or encounter record: missing latitude or longitude, precision below the 10‐min block level, evidence of transport by humans (i.e. ‘transported’, ‘rehabbed’) or a recovery code indicating a long delay between death and discovery. We removed nine records from Alaska that were spatially disjunct from the rest of our study area. We included one banding record from Florida that may be an individual of subspecies *F. s. paulus* because dispersal distance of this bird fits within the statistical distribution of distance.

We calculated natal dispersal distance and direction from latitude and longitude with the package geosphere (Hijmans, 2016a) in the r programming language, version 3.5.3 (R Development Core Team, 2019). We categorized natal dispersal as short distance (<30 km) and long distance (>30 km). We selected 30 km as a conservative break point for LDD because the maximum dispersal distance of philopatric kestrels is ~25 km (Shields, 1982) and 92% of kestrels in nest box studies move <30 km (Jacobs, 1995; Miller & Smallwood, 1997; Steenhof & Heath, 2013). We tested whether different thresholds for LDD classification impacted our results and found that results were robust to choice of LDD threshold. The dichotomous classification of short and long distances allowed us to compare relative frequency of SDD and LDD, fit LDD distance as a continuous response in a gamma regression, and fit SDD distance as an ordinal response to reduce bias in the continuous distance data. We used an ordinal response for SDD distance with three levels reflecting the lowest level of precision recorded by the BBL (same 10‐min block, different 10‐min block and distance <20 km and different 10‐min block and distance >20 km).

We used sex reported by the bander and used banding year as natal year because we only included birds that were banded as nestlings or hatch years. We used natal latitude as a proxy for migration strategy because more northern individuals migrate farther than more southern individuals (Heath, Steenhof, & Foster, 2012). We used maximum and minimum temperature anomalies, defined as the difference in monthly maximum or minimum temperature, in degrees Celsius, from mean monthly maximum or minimum over the baseline period of 1950–1980, during different parts of the annual cycle to predict LDD frequency and distance. We included maximum temperature anomaly from May and August when kestrels are provisioned by their parents and making post‐fledging exploratory movements, respectively. We included minimum monthly temperature anomaly from May because cold springs can delay food availability; January, because winter severity can affect migration distance; and March, to test if cold temperatures during spring migration influence dispersal. We used Berkeley Earth gridded 1° × 1° resolution modelled monthly temperature anomalies and extracted values at the location and year of banding for all temperature variables (Berkeley Earth, 2017). We tested the relationship between agricultural land cover and dispersal using the percentage of agricultural land cover at the natal site, corresponding to the departure phase of dispersal, at the encounter site, corresponding to the settlement phase of dispersal, and the difference in percentages between the two sites. We calculated percentage of agricultural cover using the National Land Cover Databases (NLCD, Fry et al., 2011; Homer et al., 2007, 2015; Vogelmann et al., 2001) at 30 m × 30 m resolution with r packages raster and rgdal (Bivand, Keitt, & Rowlingson, 2017; Hijmans, 2016b). We considered all classifications in the ‘Planted/Cultivated’ categories to be agricultural and all other classifications non‐agricultural. We computed percentage of agriculture in 4 square km areas, corresponding to typical kestrel home range size (Bird & Palmer, 1988). NLCD classifications exist for four discrete time periods (1992, 2001, 2006 and 2011), so we used the database closest to the year of each banding record to assign values. For banding records in Canada (*n* = 26), we assigned median values for all land cover variables because the NLCD does not cover this region and so that we could use these records in analyses, and verified that this did not affect parameter estimates by running models with agriculture predictors with and without the Canadian records. We represented relative population density with stratum‐specific annual relative abundance indices (McCaslin & Heath, 2020) based on Breeding Bird Survey data (Pardieck, Ziolkowski, Lutmerding, & Hudson, 2018), where strata are defined as the intersection of states and Bird Conservation Regions, divided by stratum area to obtain annual estimates of relative kestrel density adjusted for Breeding Bird Survey sampling bias. We considered density estimates at the natal location as a correlate of frequency and distance of dispersal. We did not have density estimates for some dispersal records (*n* = 46) because density estimates were not calculated for strata in which kestrels were detected on fewer than four BBS routes or for year‐stratum combinations with no kestrel detections.

We described the distribution of dispersal directions using wind rose diagrams and tested for uniformity of dispersal direction for SDD‐only, LDD‐only, and all distances combined using Rao spacing tests, and for differences in direction between males and females and between birds encountered alive and dead using Watson two sample tests for homogeneity in the circular package in r (Jammalamadaka & SenGupta, 2001; Lund & Agostinelli, 2007).

We modelled the relationship between intrinsic and environmental factors and natal dispersal using a hurdle model and Bayesian regression in r with Stan via rstan and rstanarm (Supporting Information S2; Carpenter et al., 2017; Goodrich, Gabry, Ali, & Brilleman, 2020; Stan Development Team, 2017). We estimated the frequency of LDD by modelling the binomial outcome of short‐ or long‐distance disperser with predictors sex, latitude, percentage of agricultural land cover (natal site, encounter site and difference between the two), natal year, temperature, relative population density at the natal site, and interactions between sex and latitude, temperature, and agriculture, and between latitude and year (Tables S4 and S5). We expected that the chance nature of band encounters, differences in the types of encounters reported by researchers and the public and inconsistencies in encounter location reporting would influence the distributions of dispersal distance, so we also included encounter condition (alive or dead) and who encountered an individual (researcher or public) to account for possible sampling bias, and a random effect of categorical natal year to control for temporal heterogeneity. We modelled the dispersal distance of LDD individuals with a gamma distribution and fit Bayesian generalized linear models with the same set of predictors and the random effect as in the frequency models (Tables S7 and S8). We modelled SDD distance using an ordered logistic (ordinal) regression for three ordinal categories with the same set of predictors (Table S9). We standardized all continuous variables prior to analysis. Correlations between covariates were <0.3 (Pearson's correlation coefficient), suggesting that multicollinearity is unlikely.

We followed current best practice for Bayesian multiple regression and used weakly informative, normally distributed priors with mean 0 and standard deviation 2.5 for all regression parameters (McElreath, 2016). In the ordinal regression, we specified priors such that the prior mean for *R* ^2^ = 0.5 and each of the three ordinal levels were equally probable under the prior (additional details about priors are shown in Supporting Information S4). We ran models for four MCMC chains with 1,000 iterations per chain (plus 1,000 iterations burn‐in), and diagnosed Markov Chain convergence using r‐hat <1.1 and by visually checking chain blending.

The BBL historically maintained data with the spatial precision of a 10‐min block of latitude and longitude and began accepting and saving records at this precision, 1‐min block precision or exact precision in the early 2000s. We ran LDD distance and frequency analyses at the reported precision (exact, 1‐min block, 10‐min block) and at the 10‐min block precision for all records to check that differences in precision between records did not bias the calculated distances. We used the original precision for each model in final models because running models at the 10‐min block precision did not influence model results. We found the most informative representation of temperature (month and min or max) and percentage agriculture (natal, encounter or difference) by comparing models for each variable (Tables S3 and S6). Then, we used the covariate for the top model of temperature and agriculture in models for frequency and magnitude. We ran models including density with a reduced number of records because some observations were missing density estimates. If there was not support for density, we removed the density variable and re‐ran the models with the full sample size. We selected best models using expected log posterior density (ELPD) with the r package loo (Vehtari, Gabry, Yao, & Gelman, 2018). Expected log posterior density is a leave‐one‐out approximation of out‐of‐sample predictive fit, and it is efficiently implemented in Stan and the loo package to avoid having to compute the density separately for each observation (Vehtari, Gelman, & Gabry, 2017). We considered information from the 95% credible intervals of covariates in equally competitive models to evaluate the direction and strength of effect.

We ran the most supported model for LDD frequency and the top model for LDD distance with the natal location as a spatial random effect using a stochastic partial differential equation (SPDE) approach in the r package inla (Lindgren & Rue, 2015; Rue, Martino, & Chopin, 2009) to test for spatial autocorrelation. We did not include a spatial random effect in the models for SDD distance because we expected the spatial effect to arise due to differences in sampling between within and outside of study areas, which is not a concern for short‐distance only movements. We compared INLA models with and without the spatial random effect to determine if spatial autocorrelation present, and if there was evidence for spatial autocorrelation, we re‐ran the full model set for that response with and without the spatial random effect in INLA. We compared models with and without spatial random effects, and selected the best INLA model using the log pseudo‐marginal likelihood (LPML), which is the sum of the log conditional predictive ordinates (CPO) to determine if spatial autocorrelation was present (Lindgren, Rue, & Lindstrom, 2011). Like ELPD, LPML is also based on the leave‐one‐out predictive distributions for each observation (Hooten & Hobbs, 2015), and it is implemented efficiently in INLA (Held, Schrödle, & Rue, 2010). If spatial autocorrelation was not present in the response, we used the most supported Stan model for inference. While we used different metrics to compare the INLA and Stan models, we emphasize that LPML and ELPD are conceptually similar with a foundation in estimating leave‐one‐out predictive distributions.

## 3 RESULTS

**Figure 1:**
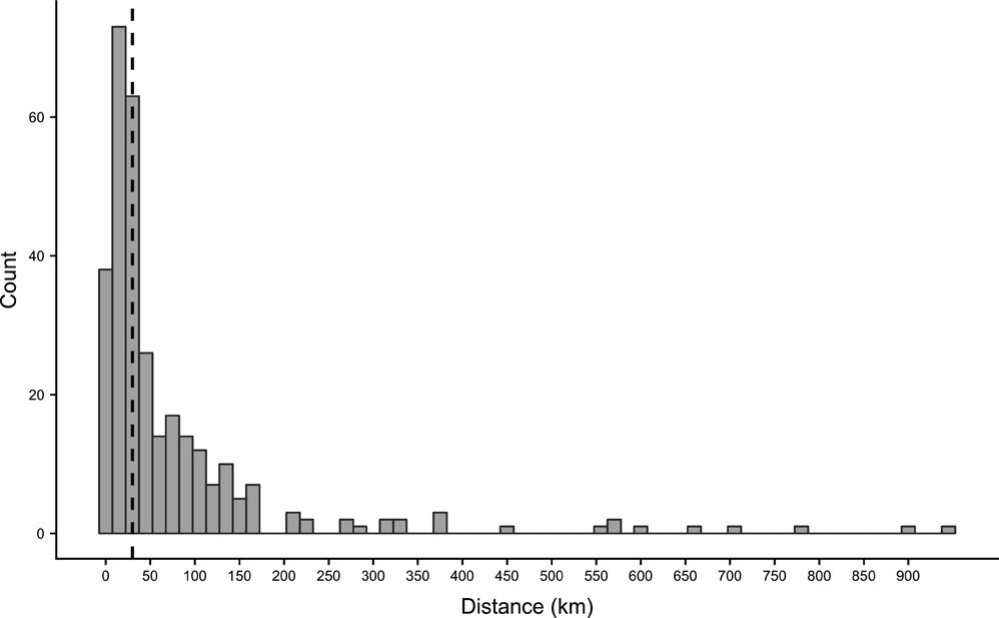
Frequency of natal dispersal distances of American kestrels from North American banding and encounter data, 1961–2015. Of 311 total individuals (161 females, 105 males, 45 unknown), 152 (86 females, 49 males, 17 unknown) dispersed a distance greater than 30 km, indicated by the dashed line

**Figure 2:**
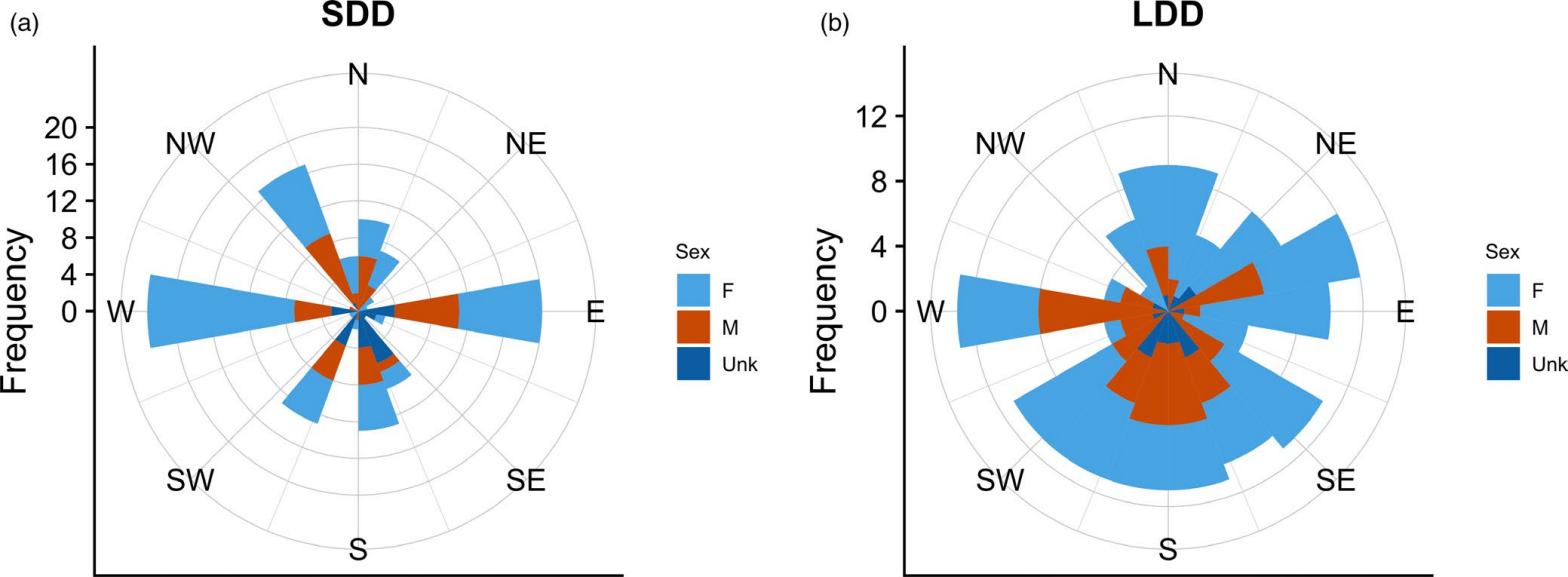
Frequency of natal dispersal directions of American kestrels from North American banding and encounter data, 1961–2015. Length of bar corresponds to frequency of direction. Dispersal direction was not uniformly distributed across all distances (*p* < 0.001), (a) short distances (*p* < 0.01) or (b) long distances (*p* < 0.01). There was no difference between sexes (*p* > 0.1) or between birds encountered alive or dead (*p* > 0.1)

Our final dataset included banding and encounter records for 311 individuals (161 females, 105 males, 45 sex unknown) banded between 1961 and 2015. Median dispersal distance for all individuals was 28.2 km and within the categories SDD and LDD, median distances were 16.4 and 87.4 km, respectively (Figure 1). Long‐distance dispersal made up 48.9% of dispersal movements (86 females, 49 males, 17 sex unknown). Within SDD, 40 individuals dispersed within a single 10‐min block, 69 individuals dispersed outside of their natal block but <20 km, and 50 individuals dispersed between 20 and 30 km. Dispersal direction was not uniformly distributed for all individuals (*p* < 0.001), SDD‐only (*p* < 0.01) or LDD‐only (*p* < 0.01). Short‐distance dispersal movements occurred more frequently in east and west directions, and LDD tended to be in southward directions (Figure 2). Dispersal directions did not differ significantly between males and females (*p* > 0.1) or alive and dead encounters (*p* > 0.1).

**Figure 3:**
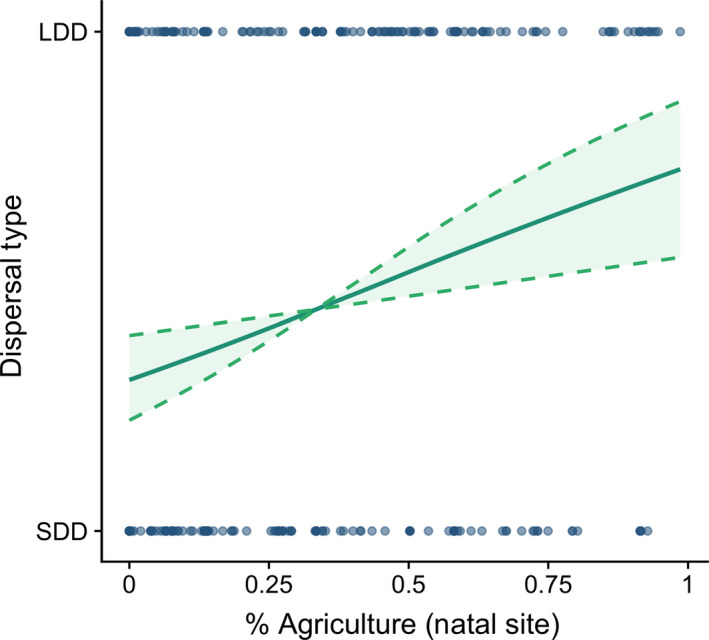
Relationship between the percentage of agriculture at natal site and long‐distance dispersal frequency in American kestrels in the United States and Canada from 1961 to 2015 from banding and encounter data. Solid line is mean predicted effect and dashed lines represent 95% credible intervals for model predictions

Relative frequency of LDD compared to SDD was best predicted by the percentage of agricultural coverage at the natal site, encounter condition and a spatial random effect of natal location (Table 2; Table S5). Percentage of agriculture was positively correlated with the likelihood of being a long‐distance disperser, with a mean increase in probability of LDD of 13% associated with increasing agricultural cover from 10% to 40% (Figure 3). The likelihood of an individual being a long‐distance disperser increased by about 20% if the bird was encountered dead, suggesting an effect of encounter sampling on patterns in the data. There was some evidence that minimum March temperature was positively correlated with frequency of long‐distance dispersal (80% of posterior marginal samples >0), but the 95% credible interval for the parameter crossed zero (95% CI −0.01 to 0.55). Frequency of LDD versus SDD was spatially autocorrelated in areas surrounding nest box study areas, indicating that short‐distance dispersers are more frequently encountered in these areas, likely because of sampling in study areas (Figure S2). There was no evidence for an association between LDD frequency and population density, sex, natal year or latitude in the most supported model.

**Table 2:** Summary of predictions, model terms and corresponding results for LDD frequency, SDD distance and LDD distance. Results indicated with (^a^) are model terms that appeared in less parsimonious but equally competitive models, suggesting there is some evidence that they may be important covariates. Credible intervals are on the standardized covariate scale so they have the correct relationship to 0

Prediction	Model term	Result (95% credible intervals)
Females will disperse farther than males	Sex	SDD (−1.17, 0.15)^a^ No support in Freq. or LDD
Migratory individuals will disperse farther than nonmigratory individuals	Natal latitude	LDD (0.12, 0.34) SDD (−0.56, 0.080)^a^ No support in Freq.
Temperature Hatching and post‐fledging max temperatures positively correlated Winter and nest‐establishment min temperatures negatively correlated	Temp	LDD, Max Aug temp × male (0.14, 0.63) Freq, Min Mar temp (−0.011, 0.55) No support in SDD
Agriculture negatively correlated with distance	Ag	LDD, Diff. in % ag (−0.30, −0.059) Freq, % ag at natal site (0.18, 0.86) No support in SDD
Environmental change over time may lead to temporal trends	Natal year	SDD (−0.39, 0.29)^a^ No support in Freq. or LDD
Sex‐bias and migratory strategy will have an interactive effect on dispersal distance	Sex × latitude	No support
Males and females may respond differently to temperature	Sex × temperature	LDD dist, Max Aug temp × male (0.14, 0.63) No support in Freq. or SDD
Migratory individuals will increase dispersal over time more than nonmigratory	Latitude × year	No support
Natal density positively correlated with LDD dispersal frequency	Population density index	SDD (−0.21, 1.09) LDD (−0.13, 0.24)^a^ No support in Freq.
Sampling will affect dispersal distances observed	Encounter condition	Freq, dead (0.38, 1.51) LDD, dead (0.10, 0.58) SDD, dead (−1.3, 0.031)

Abbreviations: LDD, Long‐distance dispersal; SDD, shorter‐distance dispersal.

^a^ Parameters from less‐parsimonious, equally competitive models.

**Figure 4:**
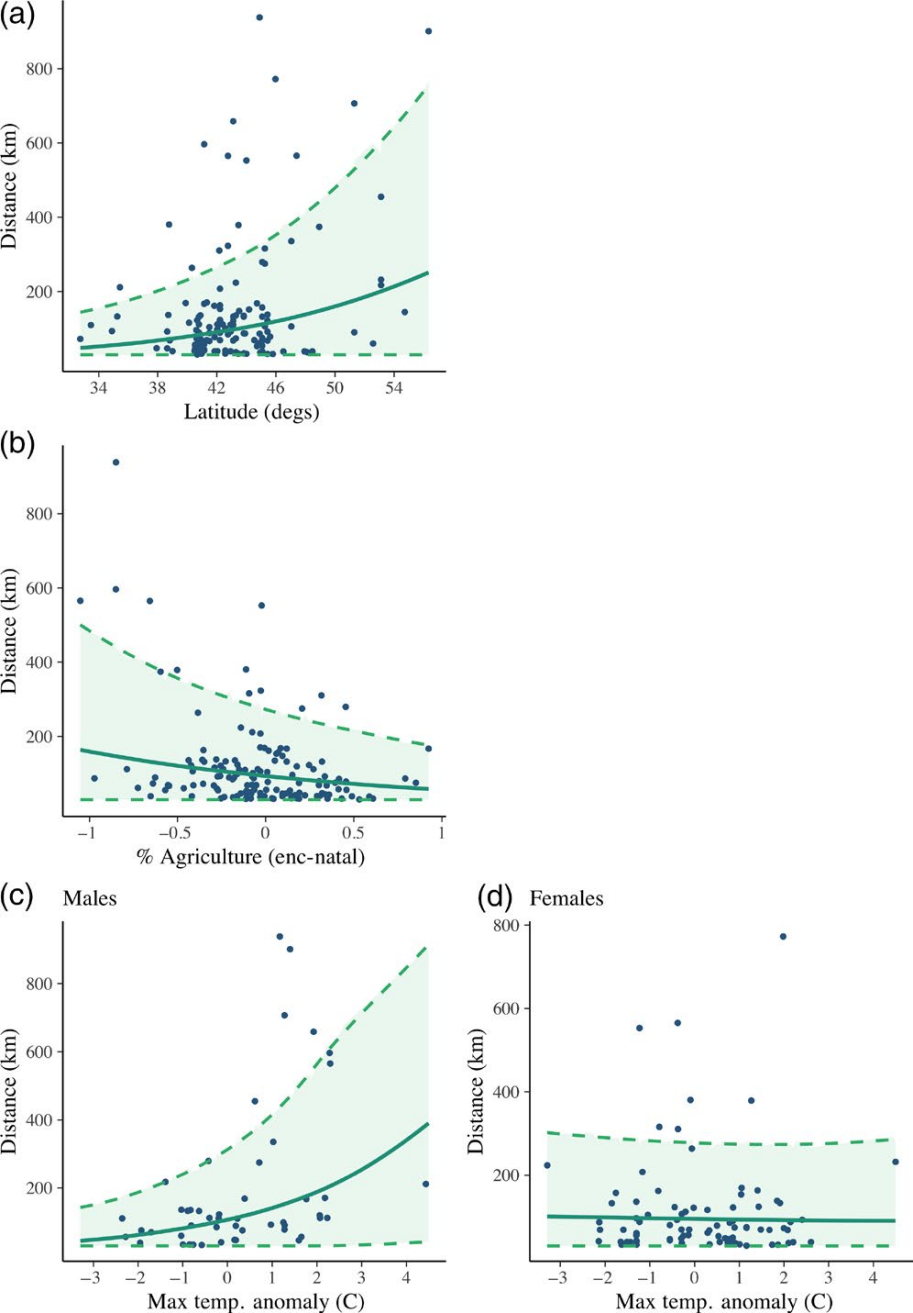
Association between latitude (a), agriculture (b), and maximum August temperature in males (c) and in females (d) on long‐distance dispersal (LDD) distance in American kestrels in the United States and Canada 1961–2015 from banding and encounter data. Solid lines are mean predicted effects and dashed lines represent 95% credible intervals for model predictions

The most supported model for LDD distance contained an interaction of sex and maximum August temperature, natal latitude and the difference in percentage of agriculture between sites (Table 2; Tables S7 and S8). For long‐distance dispersers, maximum August temperature was positively correlated with dispersal distance in males (Figure 4), but in females there was no relationship between dispersal distance and maximum August temperature. In males, an increase from average maximum August temperature to 1°C warmer than average corresponded with a predicted increase in LDD distance of 26.1 km. Latitude was positively correlated with LDD distance, and an increase in latitude from 35° to 45° was associated with a predicted 40.4 km increase (median posterior prediction) in LDD distance (Figure 4). The difference in percentage of agriculture between the encounter and natal locations was negatively correlated with distance. Thus, individuals dispersing the shortest distances were moving from relatively lower to higher percentage agriculture, those dispersing mid‐distances were moving between relatively similar percentages of agriculture, and those dispersing the greatest distances were moving from relatively higher to lower percentage agriculture (McCaslin, 2019, Figure S1.5). Increasing the percentage of agriculture at the encounter site by 25% while holding the percentage at the natal site constant resulted in a predicted 9.0 km decrease in LDD distance. Encounter condition was an important variable for LDD distance. The probability of encountering a long‐distance disperser dead rather than alive increased with distance from natal site (Table 2). There was no evidence of spatial autocorrelation in LDD distance. The top model for LDD distance was equally competitive when density was included, but the 95% credible interval for the parameter was centred on zero, indicating little evidence for correlation between density and distance (Table 2; Table S7).

For SDD, there were several equally competitive models, and the most parsimonious model suggested SDD was correlated with population density and encounter condition (Table S9). Short‐distance dispersers (<30 km) dispersing within a single 10‐min block were 20% less likely than those dispersing to a different block but going <20 km, and there was a 0.65 probability that a short‐distance disperser dispersed <20 km versus dispersing between 20 and 30 km. Population density was positively associated with SDD distance (mean log of odds = 0.6, 95% CI 0.2–1.1), corresponding to an 85% increase in odds of dispersing farther for a one standard deviation increase in population density. Within SDD, birds recaptured alive were more likely to be encountered farther from banding sites compared to dead bird encounters. Latitude, sex and year appeared in equally competitive models for SDD; in particular, there was evidence that males are 40% less likely to disperse greater distances relative to females (Table 2). However, odds ratios for latitude and year were near one, suggesting little evidence for associations between these variables and a change in SDD distance. The multiple equally competitive models for SDD distance may be due to the challenges of separating out effects using ordinal rather than continuous data for dispersal distance (Taylor, West, & Aiken, 2006).

## 4 DISCUSSION

We studied natal dispersal in American kestrels over a large spatiotemporal scale using bird banding and encounter records. Our continental‐scale approach indicated a higher frequency of LDD than previously reported using local‐scale nest box studies of kestrel dispersal. The percentage of agricultural cover was positively associated with frequency of LDD, suggesting that land cover type around the natal site influences post‐fledging movement. Additionally, we found that the latitude of the natal site, a proxy for migration strategy and the temperature during late summer predicted distance of LDD movements, indicating that LDD is associated with both intrinsic and environmental factors. However, we did not find sex‐bias in either LDD frequency or distance as reported in previous studies of SDD, and found evidence that population density was correlated with SDD but not LDD distance. Together, these results suggest that the same factors do not predict both LDD frequency and distance in kestrels, and that LDD is not driven by the same cues underlying SDD. This study provides evidence that short‐ and long‐distance dispersal may be distinct processes in kestrels and that LDD should not be summarized as extreme events occurring in the tail of a single, mostly short‐distance, dispersal distribution.

Long‐distance movements made up nearly half of all natal dispersal movements within banding records. The relatively high frequency of LDD is supported by demographic studies that report a high proportion of immigration into study populations (Brown & Collopy, 2013; Steenhof & Heath, 2013, C.J.W. McClure, unpubl. data) and analyses that show relatively low genetic structure in American kestrels even with the use of high‐resolution approaches (Brinkmeyer, 2018). We found no other studies comparing relative frequencies of short‐ and long‐distance dispersal in birds. Although large‐scale banding data may overestimate the frequency of LDD because banders do not always report encounters of their own bands within the same 10‐min block, previous studies have shown that observed patterns of dispersal are scale‐dependent (Morton et al., 2018) and that local studies of avian dispersal can yield dispersal distances an order of magnitude smaller than those observed via other methods (Tittler, Villard, & Fahrig, 2009). Thus, it is important to recognize that the true frequency of LDD probably lies somewhere between what has been reported via nest box studies and what is found across a large scale, and as our ability to track animals over large distances continues to improve and increases the possible scale of observation, we expect that empirical studies of dispersal may begin to detect higher frequencies of LDD.

The percentage of agriculture in the natal site was positively correlated with an individual's likelihood of being a long‐distance disperser, which is the opposite of our prediction (Table 2). This could be because agricultural land cover is associated with high prey abundance and open landscapes for foraging (Smallwood, 1987; Smallwood, Winkler, et al., 2009), so nestlings were well provisioned and capable of moving longer distances after fledging. This is similar to the relationship observed between body condition and dispersal distance in Spanish imperial eagles (*Aquila adalberti*) (Ferrer, 1993, Ferrer & Morandini, 2017) and Eurasian eagle owls (*Bubo bubo*) (del Mar Delgado et al., 2010), in which better nourished juveniles dispersed earlier and moved farther than poorly fed juveniles. Alternatively, areas with high percentage of agricultural land cover could attract high densities of nesting kestrels (Touihri et al., 2019), so individuals disperse from these areas to avoid competition, although we did not find correlation between relative population density and percentage of agricultural cover. Additionally, because there is evidence that dispersal distance is correlated with parental dispersal distance in kestrels (Steenhof & Heath, 2013), this pattern could arise because parents with greater dispersal ability select higher quality habitat than kestrels that disperse shorter distances.

We found a positive relationship between August maximum temperatures and dispersal distance in male kestrels, suggesting that environmental conditions during the post‐fledging period in late summer are important for driving LDD. This result supports other studies that have found natal dispersal in raptors occurs during this period, when juvenile birds explore the area surrounding their natal site prior to settlement or fall migration (Soutullo, Urios, Ferrer, & Peñarrubia, 2006; Walls & Kenward, 1995). This relationship between temperature and dispersal could arise because individuals are responding to proximate environmental cues during dispersal movements, or because warmer temperatures create more favourable conditions for flight and allow for efficient long‐distance movements (Hernández‐Pliego, Rodríguez, & Bustamante, 2015; Hernández‐Pliego, Rodríguez, Dell'Omo, & Bustamante, 2017). Walls et al. (2005) found that temperatures and wind directions during this time were strongly correlated with the onset and distance of dispersal in common buzzards, with southward winds predicting dispersal movements and dispersal distance positively correlated with winds to the west. We also found a southward trend in LDD movements, and a similar pattern has been found in Eurasian eagle owls in which the majority of individuals dispersed in the west‐southwest direction throughout the exploratory phase following fledging, apparently influenced by wind directions (del Mar Delgado et al., 2010). We did not find similar trends in SDD orientation, perhaps because either short‐ and long‐distance dispersers are not dispersing simultaneously and therefore subject to different wind, or are the result of different phenotypes responding differently to proximate environmental cues (Camacho, Martínez‐Padilla, Canal, & Potti, 2019).

The effect of sex depended on August maximum temperature, with differences between sexes only occurring at higher temperatures when males dispersed farther than females. This may be because warmer temperatures reduce the costs of LDD to a greater extent in males, either directly by allowing smaller‐bodied individuals to more efficiently move greater distances because kestrels are sexually dimorphic and males are smaller than females (Smallwood & Bird, 2002) or indirectly by influencing young males' ability to acquire a territory (Perrin & Mazalov, 1999). We found evidence of a trend towards female‐bias in SDD, and it has been well documented that female kestrels disperse farther than males in other short‐distance dispersal studies (Jacobs, 1995; Smallwood & Bird, 2002; Steenhof & Heath, 2013). Thus, our finding that LDD does not appear female‐biased suggests that SDD and LDD may be influenced by different mechanisms. Inbreeding avoidance is typically cited as the primary driver of sex‐biased dispersal in vertebrates, with the mechanism being that if one sex regularly disperses farther than the other, siblings will not interbreed (Bowler & Benton, 2005). Because we did not find sex‐biased dispersal in kestrels at a large scale, independent of temperature effects, it is possible that inbreeding avoidance is not an ultimate driver of LDD in kestrels. In a population of great tits (*Parus major*) that displayed female‐biased dispersal distance, rates of dispersal between low‐ and high‐quality habitat were not sex‐biased, suggesting the effect of sex on dispersal may be scale‐dependent (Verhulst, Perrins, & Riddington, 1997). If SDD and LDD are driven by different ultimate factors, there may also be distinct dispersal phenotypes in kestrels, similar to differences in behavioural boldness between ‘movers’ and ‘stayers’ in killifish (*Rivulus hartii*) (Fraser, Gilliam, Daley, Le, & Skalski, 2001) and phenotypic differences between dispersers and residents within several other vertebrate species (Clobert, Le Galliard, Cote, Meylan, & Massot, 2009).

Long‐distance dispersal was longer but not more frequent at higher latitudes. Therefore, long‐distance dispersers from migratory populations dispersed greater distances than those from partially migratory or resident populations, but LDD was maintained at similar frequencies in populations regardless of spatial location. This is consistent with Sutherland et al. (2000) who found that among species, migratory strategy is correlated with maximum dispersal distance but not median distance, which is determined by relative frequencies of SDD and LDD. Thus, there may be spatial variation in ability to adapt to global change if the ability to move long distances is important for adaptation, as suggested by Barbet‐Massin, Thuiller, and Jiguet (2011) who showed that future breeding ranges for several European bird species under predicted climate change is strongly influenced by mean natal dispersal distance.

We found that LDD was shortest for individuals moving from low to relatively higher percentage of agricultural cover, in which case land cover heterogeneity around the natal site was high and allowed individuals to locate areas likely to have high‐quality foraging at relatively short distances. Individuals that dispersed to areas of similar extent of agriculture relative to their natal site dispersed farther, in agreement with theory that predicts that dispersal distance should increase as spatial variation in habitat quality decreases because individuals must move farther to find substantially higher‐quality habitat (Lowe, 2009). We found that individuals dispersing from relatively high to relatively low percentage of agriculture moved the greatest distances, which is not explained by theory, but could be the result of these individuals searching for better quality habitat and ultimately reaching a threshold associated with the energetic costs of dispersal that forces them to accept lower quality habitat (Bonte et al., 2012).

Population density at the natal site was positively correlated with distance in short‐distance dispersers, suggesting that density dependence may be an important factor in dispersal at this scale. Competition for nest sites may drive increased dispersal distances in young birds that remain relatively close to natal sites. Interestingly, we did not find support for similar patterns in LDD frequency or distance, suggesting that density dependence may change with the scale of study (De Bona et al., 2019) and other factors are more closely correlated with long‐distance dispersal. However, our metric of population density was relatively coarse (Table S2), which may limit our ability to detect relationships between density and dispersal.

Encounter condition was an important predictor for frequency and distance of LDD and SDD. Individuals encountered dead were more likely to be long‐distance dispersers and, for SDD, dispersed shorter distances relative to live encounters. The association with LDD distance was the opposite, with dead encounters to be more likely at farther distances than live encounters. The higher frequency of live recaptures occurring at short distances occurs because birds that disperse short distances may remain within study areas where there is effort to capture and band birds so they are more likely to be recaptured alive in a nest box than those who disperse out of study areas. Additionally, the uneven spatial distribution of nest box studies increases the likelihood of capturing SDD in these areas, but because long‐distance movements exceed the size of study areas, observed distances are not affected by their distribution, which is likely the reason that relative frequency of LDD and SDD was spatially autocorrelated while LDD distance was not. Similar sampling effects have been found in previous studies using bird banding data to infer movement patterns (Royle & Dubovsky, 2001; Thorup, Korner‐Nievergelt, Cohen, & Baillie, 2014). Paradis et al. (1998) demonstrated the potential for bird banding data to be applied over large scales to study avian dispersal, but heterogeneity of encounter probability is a concern with using banding data in large‐scale studies (Thorup et al., 2014; van Noordwijk, 1995). For example, without accounting for the higher probabilities of dead encounters as distance increases, it is possible to confound sampling bias with a signal that LDD is more ‘risky’ than SDD. While existing banding data is a cost‐effective and powerful tool, it is important that future work with these data incorporate models that can account for encounter heterogeneity, and it would be worthwhile to collect small‐scale data on encounter probability with future bird banding analyses to parameterize models.

We were not able to account for all factors that may affect dispersal in this study. Body condition is an important intrinsic factor that can influence the length, rate and timing of dispersal (Ferrer, 1993; del Mar Delgado et al., 2010) and can alter the distribution of natal dispersal (Ferrer & Morandini, 2017). Studies of avian dispersal have often reported individuals in better condition disperse farther (Barbraud, Johnson, & Bertault, 2003; del Mar Delgado et al., 2010; Ferrer, 1993; Ferrer & Morandini, 2017; Møller et al., 2006). Alternatively, perhaps dependent on landscape configuration, competition for nest sites may force individuals in poorer physical condition to disperse farther (Gauthreaux, 1978; Waser, 1985). Additionally, dispersal propensity may have a genetic basis (Forero, Donázar, & Hiraldo, 2002; Saastamoinen et al., 2018; Steenhof & Heath, 2013). Unfortunately, we did not have data on body condition or parental dispersal to address these factors. Hopefully, continuing to improve the data collected for banded or tracked individuals will allow us to address whether these intrinsic factors may interact with the environmental to influence dispersal.

We found a high frequency of LDD and a response of LDD to intrinsic and environmental factors that together suggest that long‐distance dispersal in American kestrels may be a distinct process from short‐distance dispersal. We illustrated that studies at different scales capture different frequencies of LDD in kestrels and show that different factors play distinct roles in LDD and SDD. To our knowledge, this is the first evidence that long‐distance and short‐distance dispersal are different phenotypes in an avian species, and highlights the need for more research designed with long‐distance movements in mind, to improve our understanding of the frequency of LDD and the drivers and dynamics of dispersal overall. Because LDD ability is an important factor for adaptation to global change via connecting populations and increasing gene flow (Barbet‐Massin et al., 2011; Greenwood, 1980), it is plausible that across taxa, LDD becomes more frequent with selective pressure for individuals to move greater distances (Kokko & López‐Sepulchre, 2006; Lowe & McPeek, 2012). Thus, it is important that ecologists designing and conducting field studies consider the possibility that long‐distance dispersal may be a distinct phenotypic process from short‐distance dispersal. Given the potential implications of long‐distance dispersal on population dynamics, it is important we strive to better understand its causes and consequences to further develop our concept of adaptation and response to global change.

## AUTHORS' CONTRIBUTIONS

H.M.M. and J.A.H. conceived the study idea and design; H.M.M., J.A.H. and T.T.C. analysed data; H.M.M., J.A.H. and T.T.C. wrote the manuscript.

## Supporting information

Supplementary Material

## Data Availability

The datasets generated and analysed during this study available at https://doi.org/10.18122/bio_data/3/boisestate (McCaslin, Caughlin, & Heath, 2019).
