# Supplementary material for: Long‐distance natal dispersal is relatively frequent and correlated with environmental factors in a widespread raptor
Source: J Anim Ecol. 2020 Jul 13;89(9):2077–88. doi: 10.1111/1365-2656.13272 (PMC7540595; doi:10.1111/1365-2656.13272)
Supplement: Supplementary file 1 — Supplementary Material [file JANE-89-2077-s001.pdf]

Supplemental Material: McCaslin, H.M., T.T. Caughlin, and J.A. Heath. 2020. Long-distance natal dispersal is relatively frequent and correlated with environmental factors in a widespread raptor. *Journal of Animal Ecology.*

S1 Letter regarding short-distance dispersal analysis

Both reviewers suggested we add an analysis of short-distance dispersal distances to our manuscript of correlates of long-distance dispersal distances, to improve comparisons. In our first version, we did not include an analysis of short distances because of limitations in how bird banding data has been historically collected and recorded. Banding and encounter locations (latitude and longitude) can be reported as exact precision, 1-minute block precision, or 10-minute block precision (Gustafson, Hildenbrand and Metras. 1997. The North American Bird Banding Manual (Electronic Version). Version 1.0). Observations recorded at the one-minute and ten-minute block precisions are assigned the latitude and longitude of the center of the block in which the observation took place. In North America, 10 minutes of latitude is around 18.5 km on average and 10 minutes of longitude is approximately 14.6 km. Therefore, if both banding and encounter locations are recorded at the 10-minute block level, the distance recorded will be 0 even though the bird could have dispersed up to about 24 km if it moved corner to corner, and a bird that dispersed less than a kilometer could be recorded as moving 18.5 km if it crossed the boundary between two blocks. Technology and the precision levels at which the BBL has encouraged contributors to record data have changed throughout time so the database contains a mix of the different precision levels, even among locations for a single bird (i.e. banding location may be recorded at 10-minute level and encounter with exact precision, leading to a distance that could be shorter or longer than the true movement). For this reason, among observations of distances less than 30 km, there are more zeros than would be expected otherwise, because many records are at the 10-minute block level and both banding and encounter are recorded at the center of the block, and more problematically, the error associated with using multiple precision levels can be the same magnitude as the true movements themselves, making it nearly impossible

to separate error from the actual data. This problem exists in the long distance dispersal (LDD) as well, but in this case, the error is smaller than the movements studied, and we analyzed data using the recorded precisions but also using 10-minute block precision across all records to ensure that differing precisions did not influence the LDD results.

We understand the reviewers' perspective about the value of comparing short distance in this paper, so we have included an analysis of the SDD data from the BBL database in the updated manuscript. Because of the data quality concerns explained above, we used an ordered logistic regression with three distance categories that correspond to the number of blocks moved to examine the correlates of SDD. We found an association between SDD and density and evidence of female-bias in dispersal distances that is consistent with previous studies of SDD.

S2 Analysis Workflow and Model Details

We modeled dispersal in American kestrels using a hurdle model in a Bayesian framework. We modeled the relative frequencies of long-distance dispersal (LDD) and short-distance dispersal (SDD) using a logistic regression. Then, we modeled LDD distance using a gamma regression. Due to data limitations, we modeled SDD distance as an ordinal response with an ordered logistic regression. Our full modeling approach is shown in (1)-(5), and our full workflow for selecting predictors and including spatial random effects is detailed in Fig. S1.

$$\text{dist}_{LDD} \sim \begin{cases} 0, & LDD = 0 \\ \text{Gamma}(a, \frac{a}{\mu}), & LDD = 1 \end{cases} \tag{1}$$

$$LDD \sim \text{Bernoulli}(p) \tag{2}$$

$$\text{logit}(p_i) = \boldsymbol{\alpha}\mathbf{X}_\alpha + \tau_{yr} \tag{3}$$

$$\log(\mu_i) = \boldsymbol{\beta}\mathbf{X}_\beta + \tau_{yr} \tag{4}$$

$$\text{P}(SDD \leq k) = \text{logit}^{-1}(\theta_0 + \sum \boldsymbol{\theta}\mathbf{X}_\theta) \tag{5}$$

We modeled each response with predictors sex, latitude, percentage of agricultural land cover (natal site, encounter site, and difference between the two), natal year, temperature, relative population density at the natal site, and interactions between sex and latitude, temperature, and agriculture, and between latitude and year. In each model set, we included encounter condition (alive or dead) or who encountered an individual (researcher or public)) to account for possible sampling bias. For each response, we selected the most-predictive set of covariates ($\mathbf{X}_\alpha$, $\mathbf{X}_\beta$, and $\mathbf{X}_\theta$). The logistic and gamma regressions included a random effect of year to control for temporal heterogeneity.

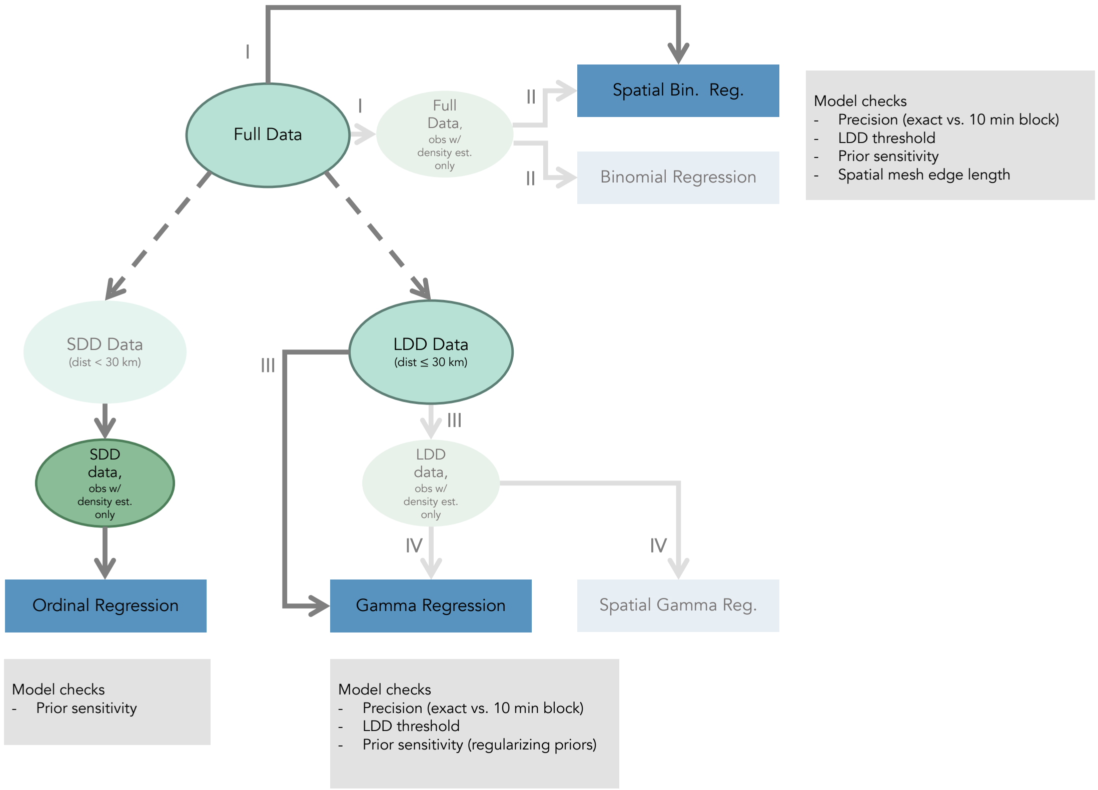

Figure S1: Flow chart outlining data and modeling approach. Bold arrows and polygons indicate final modeling scheme used for inference; ovals indicate datasets, and rectangles indicate models. Gray rectangles outline additional checks we did at each model stage to ensure results were robust to factors like LDD/SDD threshold, priors, and record precision. Each numbered pair of arrows indicates point in the analysis workflow where we used a model selection metric (LMPL or ELPD) to select the most-predictive covariate or model-type. For example, at pair I, we fit the model using the subset of individuals for which we had density estimates, and because LPML indicated density was not an important predictor, we refit the models using the full set of individuals, omitting density as a predictor.

S3 Covariate Descriptive Statistics

Table S1: Abbreviations for all covariates used in analysis of dispersal in American kestrels banded in North America from 1961-2015.

Abbreviation	Description
Lat	Natal latitude
Sex	Sex (male, female, unknown)
Year	Natal year
DensB	Relative population density index at natal site
AgB	Percentage of agricultural cover at natal site
AgE	Percentage of agricultural cover at encounter site
AgDiff	AgE - AgB
MinMay	Temperature anomaly for minimum May temp
MinJan	Temperature anomaly for minimum Jan. temp
MinMar	Temperature anomaly for minimum Mar. temp
MaxMay	Temperature anomaly for maximum May temp
MaxAug	Temperature anomaly for maximum Aug temp
EncCond	Who encountered an individual (researcher, public)
EncWho	Condition at encounter (alive, dead)

Table S2: Descriptive statistics for continuous covariates used to model frequency and magnitude of SDD and LDD of American kestrels banded in North America from 1961-2015. Density values are relative density indices calculated on the stratum scale (strata sizes are 'Dens strata'), from Breeding Bird Survey data (McCaslin and Heath 2020, Pardieck et al. 2018). Percentage of agriculture was calculated in 4km regions around kestrel locations from NLCD data.Temperature values are minimum and maximum monthly temperature anomalies relative to a baseline period from 1951-1980 (Berkeley Earth 2017). Covariates were standardized for analysis.

Covariate	Min	Median	Max	Std. Dev
Lat	29.25	41.75	55.08	3.12
Year	1961	2004	2015	12.70
DensB	5.2e-06	2.1e-05	4.0e-04	7.9e-05
Dens strata	2112	87202	469658	
AgB	0.00	0.27	0.99	0.28
AgE	0.00	0.31	0.99	0.28
AgDiff	-0.99	0.0	0.93	0.33
MinMay	-2.62	0.75	4.24	1.56
MinJan	-6.06	1.12	10.02	2.90
MinMar	-3.82	0.56	9.77	2.15
MaxMay	-5.11	0.46	5.36	1.87
MaxAug	-2.96	0.44	4.34	1.23

S4 Model selection

S4.1 Dispersal frequency

Table S3: Model selection results using Log Pseudo Marginal Likelihoods (LPML) to determine most predictive metric from three sets of similar covariates for logistic regression for the likelihood of long-distance natal dispersal (> 30km) by American kestrels in North America. The top covariate from each of the sets was used in the full model set for this response. All models included a spatial random effect and a random effect of banding year.

Model	LPML
**Encounter**	
EncCond	194.41
EncWho	195.06
Intercept	196.75
**% Agriculture**	
AgB	192.91
Intercept	196.75
AgE	196.83
AgDiff	197.00
**Temperature**	
MinMar	195.81
MaxAug	195.86
MinJan	196.20
Intercept	196.75
MinMay	197.46
MaxMay	197.50

Table S4: Candidate models and model selection results using Log Pseudo Marginal Likelihoods (LPML) for logistic regression for the frequency of long-distance natal dispersal ($>$ 30km)by American kestrels in North America from 1961-2015, based on a subset of total individuals (n=239) because relative population density was missing for some individuals. All models contain a random effect of natal year and a spatial random effect, except for models designated as *nonsp*. Density was not a covariate in the most predictive models, so models were rerun with all individuals and with density removed (Table S4).

Model	LPML
AgB + Lat + EncCond	150.31
AgB + EncCond	150.92
AgB + Lat + DensB + EncCond	151.25
AgB + Lat + Sex + EncCond	151.27
Ag + DensB + EncCond	151.64
AgB + Year + EncCond	151.70
AgB + Sex + EncCond	151.81
AgB + Lat + Sex + DensB + EncCond	152.00
AgB	152.34
AgB + Year + DensB + EncCond	152.61
AgB + DensB	152.94
EncCond	153.21
AgB + MinMar + EncCond	153.90
DensB + EncCond	153.92
AgB + MinMar + DensB + EncCond	154.44
Intercept	154.73
Lat	154.77
AgB + Lat + EncCond, *nonsp*	155.36
DensB	155.41
Lat + DensB	155.49
AgB + Lat + DensB + EncCond, *nonsp*	155.78
Sex	155.84
Year	155.85
AgB + MinMar + DensB + EncCond, *nonsp*	156.10
AgB + EncCond, *nonsp*	156.33
Sex + DensB	156.37
Year + DensB	156.45

Table S5: Candidate models and model selection results using Log Pseudo Marginal Likelihoods (LPML) for logistic regression for the frequency of long-distance natal dispersal (> 30km) by American kestrels in North America based on banding and encounter data from 1961-2015. The model with the lowest LPML is considered the best fitting model. All models contain a random effect of natal year and a spatial random effect, except for models designated as *nonsp*. All models with a spatial random effect outperformed all other non-spatial models.

Model	LPML
AgB + MinMar + EncCond	189.49
AgB + Lat + Sex + EncCond	190.26
AgB + Lat + EncCond	190.45
AgB + Sex + EncCond	190.84
AgB + EncCond	190.92
AgB + Lat + Sex + Year + EncCond	190.97
AgB + Lat + Year + EncCond	191.05
AgB + Year + EncCond	191.62
AgB + Sex + Year + EncCond	191.63
Sex x AgB + EncCond	191.95
AgB	192.91
Sex x MinMar + EncCond	192.95
MinMar + EncCond	193.18
Sex + EncCond	193.50
Lat + EncCond	193.76
Sex x Lat + EncCond	194.38
Lat x Year + EncCond	194.39
EncCond	194.41
Year + EncCond	194.72
MinMar	195.81
Lat	196.41
Sex	196.59
Intercept	196.75
Year	197.58
AgB + Lat + Sex + EncCond, *nonsp*	204.73
AgB + Lat + EncCond, *nonsp*	205.62
AgB + MinMar + EncCond, *nonsp*	206.26
Intercept, *nonsp*	216.50

S4.2 Long-distance dispersal distance

Table S6: Model selection results to determine most predictive metric from three sets of similar covariates for long-distance natal dispersal distance by American kestrels in North America, using expected log pointwise posterior density (ELPD). The top covariate from each of the sets was used in the full model set for this response. All models included a random effect of banding year. Dispersal distance was not spatially autocorrelated at this scale, so a spatial random effect was omitted.

Model	ELPD diff	SE ELPD diff	ELPD LOO	SE ELPD LOO	Eff Pars Loo	LOOIC
**Encounter**						
EncCond	0.00	0.00	-893.17	18.02	29.68	1786.35
EncWho	-2.99	4.53	-896.17	18.37	31.65	1792.34
Intercept	-7.08	5.18	-900.26	17.87	29.50	1800.52
**% Agriculture**						
AgDiff	0.00	0.00	-892.17	16.85	24.51	1784.35
AgE	-4.73	3.83	-896.91	17.39	26.24	1793.81
AgB	-6.63	3.63	-898.80	17.20	27.95	1797.60
Intercept	-8.08	4.87	-900.26	17.87	29.50	1800.52
**Temperature**						
MaxAug	0.00	0.00	-892.65	16.67	27.49	1785.30
MinJan	-5.81	4.21	-898.46	17.60	28.77	1796.91
MinMay	-6.29	4.41	-898.94	17.59	26.44	1797.88
Intercept	-7.61	4.18	-900.26	17.87	29.50	1800.52
MaxMay	-7.62	4.06	-900.27	17.56	29.28	1800.55
MinMar	-7.85	4.20	-900.50	17.60	28.92	1801.00

Table S7: Candidate models and model selection results for long-distance dispersal distance in American kestrels in the United States and Canada 1961-2015, based on a subset of total individuals (n=105) because relative population density was missing for some individuals. All models include a random effect of natal year. Differences in ELPDs and standard errors of ELPD difference suggests the top two models are equally-competitive, and models were run with density removed to include the full set of individuals (Table S7).

Model	ELPD diff	SE ELPD diff	ELPD LOO	SE ELPD LOO	Eff Pars Loo	LOOIC
Sex x MaxAug + AgDiff + Lat + EncCond	0.00	0.00	-604.49	13.49	21.62	1208.98
Sex x MaxAug + AgDiff + Lat + DensB + EncCond	-0.35	0.56	-604.84	13.44	22.79	1209.68
Sex x MaxAug + AgDiff + Lat + Year + EncCond	-0.75	0.55	-605.24	13.59	22.88	1210.49
Sex x MaxAug + AgDiff + Lat + DensB	-1.15	2.50	-605.64	13.67	24.97	1211.28
Sex x MaxAug + AgDiff + Lat + Year + DensB	-1.28	2.64	-605.77	13.69	25.62	1211.55
Sex x MaxAug + AgDiff + Lat + Year + DensB + EncCond	-1.53	0.66	-606.03	13.57	24.33	1212.05
Lat + MaxAug + AgDiff + Sex + EncCond	-2.16	3.14	-606.66	13.64	19.94	1213.31
Lat + MaxAug + AgDiff + Sex + DensB + EncCond	-2.84	3.05	-607.34	13.62	20.33	1214.67
Lat + MaxAug + AgDiff + EncCond	-2.90	5.49	-607.39	13.65	21.11	1214.78
Lat + MaxAug + AgDiff + Year + DensB	-3.37	5.10	-607.86	13.31	22.93	1215.73
Lat + MaxAug + AgDiff + Year + EncCond	-3.40	5.08	-607.89	13.63	21.87	1215.79
Lat + AgDiff + MaxAug + DensB + EncCond	-3.46	5.46	-607.95	13.51	21.42	1215.90
Sex x Lat + AgDiff + MaxAug + EncCond	-3.61	3.12	-608.10	13.91	22.06	1216.21
Lat + MaxAug + AgDiff + DensB	-3.61	5.35	-608.11	13.17	22.72	1216.21
Lat + MaxAug + AgDiff + Year + DensB + EncCond	-3.92	5.23	-608.41	13.60	22.81	1216.82
Lat + MaxAug + AgDiff + Sex + DensB	-4.50	3.80	-608.99	13.85	22.90	1217.99
Lat + AgDiff + EncCond	-4.58	5.98	-609.07	13.97	20.22	1218.14
Sex x Lat + MaxAug + AgDiff + DensB + EncCond	-4.59	3.17	-609.08	13.76	22.17	1218.16
Lat + EncCond	-4.97	7.18	-609.46	14.85	25.06	1218.93
Lat + AgDiff + DensB + EncCond	-5.34	5.94	-609.83	13.87	20.53	1219.65
Sex x Lat + MaxAug + AgDiff + DensB	-5.65	3.98	-610.14	14.03	23.84	1220.29
Sex x Lat + MaxAug + AgDiff + DensB	-5.65	3.98	-610.14	14.03	23.84	1220.29
Lat + DensB + EncCond	-5.84	7.33	-610.33	14.88	25.74	1220.67
Lat + DensB	-5.92	6.72	-610.42	14.01	24.96	1220.83
Lat + AgDiff + DensB	-5.98	5.92	-610.47	13.41	21.12	1220.94
Sex + EncCond	-9.33	5.24	-613.82	14.81	21.10	1227.65
Sex + DensB + EncCond	-10.29	5.23	-614.78	14.85	21.88	1229.56
EncCond	-12.08	8.23	-616.57	15.73	25.42	1233.13
Sex + DensB	-12.19	5.15	-616.68	14.48	21.55	1233.36
DensB + EncCond	-12.52	7.65	-617.01	15.34	24.60	1234.02
Year + EncCond	-13.19	8.03	-617.68	15.68	26.07	1235.36
DensB	-13.32	6.89	-617.81	14.28	22.20	1235.62
Year + DensB + EncCond	-13.49	7.64	-617.98	15.35	25.66	1235.96
Intercept	-14.02	7.42	-618.51	14.73	23.99	1237.02
Year + DensB	-14.62	7.18	-619.11	14.54	23.58	1238.22

Table S8: Candidate models and model selection results for long-distance dispersal distance in American kestrels in the United States and Canada 1961-2015. The best fitting model was selected using the Expected Log Pointwise Posterior Density (ELPD). All models contain a random effect of natal year. Comparing models with a spatial random effect using LPML indicated no spatial autocorrelation and spatial random effects were omitted from this model. This analysis with all individuals indicates the same best fitting model as the analysis on the subset of individuals with density estimates (Table S6).

Model	ELPD diff	SE ELPD diff	ELPD LOO	SE ELPD LOO	Eff Pars Loo	LOOIC
Sex x MaxAug + AgDiff + Lat + EncCond	0.00	0.00	-873.02	15.93	21.22	1746.04
Sex x MaxAug + AgDiff + Lat + Year + EncCond	-0.96	0.32	-873.98	15.93	22.68	1747.96
Lat + MaxAug + AgDiff + EncCond	-1.49	4.40	-874.51	15.60	21.29	1749.01
Sex x MaxAug + AgDiff + Lat	-2.15	3.17	-875.17	15.69	22.80	1750.34
Sex x Lat + MaxAug + AgDiff + EncCond	-2.42	3.45	-875.44	15.96	24.96	1750.89
Lat + MaxAug + AgDiff + Sex + EncCond	-2.57	3.31	-875.58	15.83	23.48	1751.17
Lat + MaxAug + Sex + EncCond	-2.57	3.31	-875.58	15.83	23.48	1751.17
Lat + MaxAug + AgDiff + Year + EncCond	-2.71	4.31	-875.73	15.90	23.21	1751.46
Sex x MaxAug + AgDiff + Lat + Year	-3.18	3.16	-876.20	16.06	24.74	1752.39
Lat + AgDiff + EncCond	-3.34	4.78	-876.36	15.65	19.97	1752.71
Sex x Lat + MaxAug + AgDiff + Year + EncCond	-3.57	3.45	-876.59	16.07	25.81	1753.19
Lat + AgDiff + Sex + EncCond	-3.60	3.15	-876.62	15.81	21.91	1753.24
Sex x Lat + AgDiff + EncCond	-3.67	3.57	-876.68	15.86	23.13	1753.37
Lat + MaxAug + AgDiff + Sex + Year + EncCond	-3.80	3.33	-876.82	15.93	24.59	1753.64
Sex x MaxAug + Lat + EncCond	-3.86	3.69	-876.88	16.52	24.67	1753.76
Lat + MaxAug + AgDiff	-4.02	5.04	-877.04	15.30	22.61	1754.08
Lat + AgDiff + Year + EncCond	-4.08	4.85	-877.10	15.64	20.74	1754.19
Lat + AgDiff + Sex + Year + EncCond	-4.35	3.12	-877.37	15.65	22.16	1754.73
Lat + Sex + MaxAug + AgDiff	-4.57	4.49	-877.59	15.66	24.26	1755.18
Sex x Lat + AgDiff + Year + EncCond	-4.67	3.60	-877.69	15.90	23.93	1755.38
Sex x MaxAug + Lat + Year + EncCond	-4.98	3.67	-878.00	16.81	26.29	1756.00
Sex x Lat + AgDiff + MaxAug	-5.49	4.81	-878.51	16.25	26.61	1757.02
Sex x Lat + MaxAug + AgDiff + Year	-5.96	4.89	-878.98	16.24	27.39	1757.97
Lat + Sex + AgDiff	-6.18	4.43	-879.20	15.68	23.04	1758.40
Sex x Lat + MaxAug + EncCond	-6.44	5.41	-879.46	16.92	29.55	1758.93
Lat + MaxAug + EncCond	-6.55	6.44	-879.57	17.19	27.22	1759.14
Lat + MaxAug + Sex + EncCond	-6.56	5.36	-879.58	16.82	27.14	1759.16
Lat + MaxAug + Year + EncCond	-6.63	6.13	-879.64	17.13	27.84	1759.29
Lat + AgDiff	-6.66	5.72	-879.68	15.41	21.43	1759.36
Sex x Lat + MaxAug + EncCond	-7.40	5.79	-880.42	17.16	31.19	1760.84
Lat + MaxAug + Sex + Year + EncCond	-7.66	5.48	-880.68	16.91	28.43	1761.36
Sex x MaxAug + AgDiff + EncCond	-9.13	4.52	-882.15	16.35	22.00	1764.30
Lat + Sex + MaxAug	-9.48	5.78	-882.50	16.31	27.59	1764.99
Lat + Sex + EncCond	-9.74	5.88	-882.76	16.87	27.24	1765.53
Lat + EncCond	-9.88	7.39	-882.90	17.30	27.71	1765.80
Lat + Sex + Year + EncCond	-10.21	5.75	-883.23	16.93	28.56	1766.47
Sex x Lat + EncCond	-10.22	6.13	-883.24	17.17	30.46	1766.48
Sex x MaxAug + AgDiff + Year + EncCond	-10.48	4.50	-883.50	16.70	24.05	1766.99
Lat + Year + EncCond	-10.83	7.32	-883.85	17.34	28.91	1767.70
Sex x Lat + Year + EncCond	-11.23	6.26	-884.25	17.24	31.46	1768.50
AgDiff + Sex + EncCond	-11.76	5.45	-884.78	16.29	22.24	1769.56
MaxAug + AgDiff + Year + EncCond	-11.82	6.56	-884.84	16.51	24.88	1769.67
Sex + MaxAug + AgDiff + Year + EncCond	-11.91	4.93	-884.93	16.32	25.58	1769.85
Sex + AgDiff + Year + EncCond	-12.56	5.45	-885.58	16.15	23.02	1771.16
Lat x Year + EncCond	-12.60	7.62	-885.62	17.74	30.61	1771.24
Sex x AgDiff + EncCond	-12.88	5.55	-885.90	16.32	23.67	1771.80
Sex x MaxAug + Year + EncCond	-13.10	5.19	-886.12	16.92	25.14	1772.24
Sex x MaxAug + EncCond	-13.25	5.57	-886.27	16.91	24.09	1772.55
Lat	-13.26	7.43	-886.28	16.35	26.81	1772.56
Lat + Sex + Year	-13.30	6.08	-886.32	16.32	28.14	1772.64
Lat + Sex	-13.34	6.12	-886.36	16.29	27.18	1772.71
MaxAug + AgDiff + EncCond	-13.62	7.71	-886.64	16.67	22.22	1773.28
AgDiff + EncCond	-14.09	7.78	-887.11	16.68	21.72	1774.23
AgDiff + Year + EncCond	-14.25	7.50	-887.27	16.67	23.14	1774.54
Sex + MaxAug + Year + EncCond	-14.93	6.08	-887.95	16.91	28.45	1775.90
MaxAug + Sex + EncCond	-15.12	6.33	-888.14	16.93	27.51	1776.29
MaxAug + Year + EncCond	-15.37	7.14	-888.38	17.33	28.96	1776.77
MaxAug + EncCond	-15.73	7.66	-888.75	17.46	29.01	1777.50
Sex + EncCond	-17.62	6.90	-890.64	17.11	26.31	1781.27
Sex + Year + EncCond	-18.91	6.84	-891.93	17.27	28.19	1783.86
EncCond	-20.16	8.99	-893.17	18.02	29.68	1786.35
Year + EncCond	-21.33	8.96	-894.35	18.30	30.28	1788.71
Sex	-23.81	7.97	-896.83	17.20	27.29	1793.66
Year	-26.58	9.62	-899.60	17.88	28.11	1799.20
Intercept	-27.24	9.97	-900.26	17.87	29.50	1800.52

S4.3 Short-distance dispersal distance

Table S9: Candidate models and model selection results for short-distance dispersal distance in American kestrels in the United States and Canada 1960-2015, using a three-level ordinal response for distance (same ten-minute-block (TMB); different TMB & distance < 20 km; different TMB & 20 km < distance < 30 km) including relative population density at the natal location as a covariate, fit to a subset of the data for which population density indices were available (n=146). Differences in ELPDs and standard errors of ELPD difference suggests multiple equally-competitive models, so the most parsimonious of these, the model including natal population density and encounter condition, was used to make inference.

Model	ELPD diff	SE ELPD diff	ELPD LOO	SE ELPD LOO	Eff Pars Loo	LOOIC
Lat + DensB + EncCond	0.00	0.00	-153.41	4.25	4.59	306.81
DensB + EncCond	-0.23	1.35	-153.63	4.00	3.67	307.26
Lat + Sex + DensB + EncCond	-0.29	1.47	-153.70	4.38	6.06	307.40
Sex + DensB + EncCond	-0.34	1.92	-153.74	4.11	5.14	307.49
Lat + Year + DensB + EncCond	-0.56	0.34	-153.96	4.18	5.12	307.92
Lat + Sex + Year + DensB + EncCond	-0.90	1.50	-154.31	4.32	6.69	308.61
Sex + DensB	-0.98	2.87	-154.39	3.87	4.27	308.78
Sex x Lat + DensB + EncCond	-1.13	1.99	-154.54	4.44	7.66	309.07
Lat + Sex + DensB	-1.31	2.70	-154.71	4.01	5.06	309.43
DensB	-1.34	2.59	-154.74	3.51	2.60	309.49
Lat + DensB	-1.62	2.33	-155.02	3.63	3.40	310.05
Lat + Sex + Year + DensB	-2.16	2.67	-155.56	4.00	5.94	311.13
Lat + Year + DensB	-2.53	2.26	-155.94	3.59	4.42	311.87
Sex x Lat + DensB	-2.84	3.06	-156.25	4.05	7.06	312.49
Lat + Sex + EncCond	-3.19	2.82	-156.59	3.98	5.43	313.19
Lat + EncCond	-3.24	2.38	-156.64	3.70	3.73	313.29
Lat + Sex	-4.02	3.57	-157.42	3.67	4.59	314.85
Sex + EncCond	-4.10	3.31	-157.50	3.52	4.60	315.01
Lat + Year + EncCond	-4.26	2.38	-157.66	3.58	4.83	315.33
Sex x Lat + EncCond	-4.46	3.09	-157.87	4.04	7.36	315.73
Lat	-4.79	3.26	-158.20	2.97	2.83	316.39
Lat + Sex + Year	-4.88	3.58	-158.28	3.64	5.50	316.56
Sex x Lat	-5.70	3.80	-159.11	3.70	6.67	318.21
Lat + Year	-5.86	3.29	-159.26	3.04	3.98	318.53

S5 Additional Model Details

S5.1 Priors

For the gamma regression of LDD distance, we used weakly informative, normally distributed priors with mean 0 and standard-deviation 2.5 for all regression priors, allowing rstanarm to adaptively adjust standard deviation for each parameter (Goodrich et al. 2020). However, results indicated priors were not moving from 2.5, indicating this was a reasonable prior choice. We also re-ran models with strongly regularizing priors (sd = 0.2) to ensure models were not overfit. Regularizing priors prevent overfitting, including multicollinearity, by ensuring that only the covariates with strongest effects impact model performance (McElreath 2016). We found that our results were robust to prior choice, including highly regularizing priors, suggesting little influence of multicollinearity in our model design.

For the ordered logistic regression of SDD distance, we specified priors such that the prior mean for R2 = 0.5 and each of the three ordinal levels were equally probable under the prior, using a Dirichlet prior on the probability of the outcome falling in each category when the predictors are held at their means (Goodrich et al. 2020). Additionally, we tested for prior sensitivity and multicollinearity in predictors by rerunning the models with a regularizing prior (R2=0.25), and with the Dirichlet priors specified so that one same-ten-minute-block outcomes were more likely than outcomes in the greater-distance categories. Again, we found that our results were robust to prior choice, including regularizing priors.

We found evidence of spatial autocorrelation in the binomial response, and fit this model using a Spatial Partial Differential Equation (SPDE) approach in INLA (Krainski et al. 2019). We used the default INLA priors for the fixed effects, with mean 0 and precision 0.001. We specified the spatial mesh with a maximum edge length of 40 km, and found the spatial range to be about 136 km. Thus, we refit the top model with a maximum edge length of 20 km, following INLA's suggestion to specify a max edge length less than 1/5 of the spatial range (Krainski et al. 2019), which we found did not affect inference.

S5.2 Spatial Autocorrelation in binomial response

Fitting the logistic regression with relative frequency of LDD and SDD with a spatial random effect indicated this response was spatially autocorrelated to a distance of 136 km (Fig S1a). Further, this autocorrelation appears strongly related to the distribution of kestrel nest box studies across the continent, particularly in southern Idaho, Wisconsin, and along parts of the East Coast of the United States (Fig S1(b)). We used the model with the spatial effect to make inference, because the SPDE accounted for the influence of proximity to nest box studies in our results.

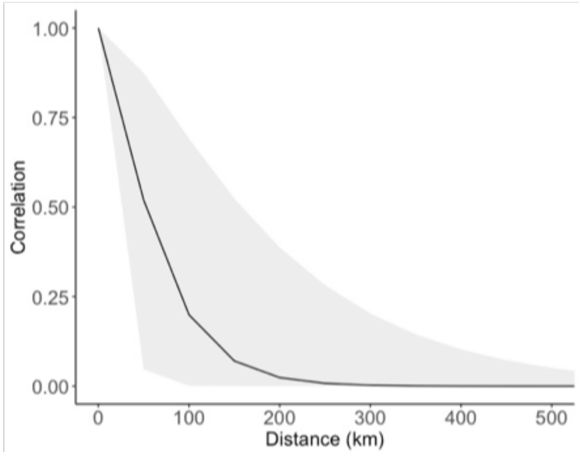

(a) Spatial autocorrelation in frequency of long-distance disperal distance.

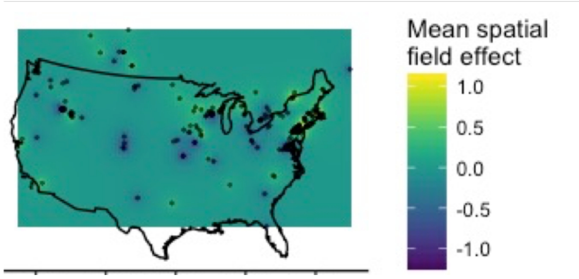

(b) Effect of space on likelihood of LDD for American kestrel banding data. Areas of strong spatial effect tend to be near kestrel nest box study areas.

Figure S2: Spatial autocorrelation in LDD frequency.

References

Berkeley Earth (2017) Gridded Data: Monthly Land (1833 – Recent). Berkeley Earth, http://berkeleyearth.lbl.gov/auto/Global/Gridded/Gridded_README.txt

Fry J, Xian G, Jin S, Dewitz J, Homer C, Yang L, ... Wickham J (2011) Completion of the 2006 National Land Cover Database for the conterminous United States. Photogramm Eng Remote Sens 77: 858-864.

Goodrich B, Gabry J, Ali I, Brilleman S (2020). "rstanarm: Bayesian applied regression modeling via Stan." R package version 2.19.3, https://mc-stan.org/rstanarm.

Homer CG, Dewitz JA, Yang L, Jin S, Danielson P, Xian G, ... Megown K (2015) Completion of the 2011 National Land Cover Database for the conterminous United States – Representing a decade of land cover change information. Photogramm Eng Remote Sens 81: 345-354.

Homer C, Dewitz J, Fry J, Coan M, Hossain N, Larson C, ... Wickham J (2007) Completion of the 2001 National Land Cover Database for the conterminous United States. Photogramm Eng Remote Sens 73: 337-341.

Krainski ET, Gomez-Rubio V, Bakka H, Lenzi A, Castro-Camilo D, Simpson D, Lindgren F, Rue H (2019) Advanced spatial modeling with stochastic partial differential equations using R and INLA. CRC Press/Taylor Francis Group. https://becarioprecario.bitbucket.io/spde-gitbook/

McElreath R. (2016) Statistical rethinking: A Bayesian course with examples in R and Stan. Boca Raton, FL: Chapman & Hall/CRC Press

McCaslin HM, Heath JA (2020) Patterns and mechanisms of heterogeneous breeding distribution shifts of North American migratory birds. J. Avian Biol. https://doi.org/10.1111/jav.02237

Pardieck KL, Ziolkowski DJ, Lutmerding M, Hudson, M-AR (2018) North American Breeding Bird Survey Dataset 1966-2017, version 2017.0. US Geological Survey Patuxent Wildlife Research Center. https://doi.org/10.5066/F76972V8. Accessed 14 May 2018.

Vogelmann JE, Howard SM, Yang L, Larson CR, Wylie BK, Van Driel JN (2001) Completion of the 1990's National Land Cover Data set for the conterminous United States. Photogramm Eng Remote Sens 67: 650-662.
